# Aminoacyl-tRNA Synthetase Complexes in Evolution

**DOI:** 10.3390/ijms16036571

**Published:** 2015-03-23

**Authors:** Svitlana Havrylenko, Marc Mirande

**Affiliations:** 1 Laboratoire d’Enzymologie et Biochimie Structurales (LEBS), CNRS, Université Paris-Sud, 1 avenue de la Terrasse, 91190 Gif-sur-Yvette, France; 2 Institute for Integrative Biology of the Cell (I2BC), CEA, CNRS, Université Paris-Sud, 1 avenue de la Terrasse, 91190 Gif-sur-Yvette, France

**Keywords:** multi-aminoacyl-tRNA synthetase complexes, supramolecular assemblies, evolution, function

## Abstract

Aminoacyl-tRNA synthetases are essential enzymes for interpreting the genetic code. They are responsible for the proper pairing of codons on mRNA with amino acids. In addition to this canonical, translational function, they are also involved in the control of many cellular pathways essential for the maintenance of cellular homeostasis. Association of several of these enzymes within supramolecular assemblies is a key feature of organization of the translation apparatus in eukaryotes. It could be a means to control their oscillation between translational functions, when associated within a multi-aminoacyl-tRNA synthetase complex (MARS), and nontranslational functions, after dissociation from the MARS and association with other partners. In this review, we summarize the composition of the different MARS described from archaea to mammals, the mode of assembly of these complexes, and their roles in maintenance of cellular homeostasis.

## 1. Introduction

Decoding of genetic information is an essential step for all living organisms. The process of translation of the genetic message contained in mRNA into proteins is a universal mechanism conserved, with minor modifications, in the three branches of the tree of life, from bacteria, archaea, and to eukaryotes. A family of enzymes, the aminoacyl-tRNA synthetases, is responsible for pairing a specific amino acid to a cognate tRNA, thus establishing a univocal relationship between a triplet of nucleotides, the anticodon, and an elementary piece of proteins. Because the proper functioning of these enzymes is essential to gene expression, extensive biochemical and structural analysis of these proteins has been conducted, leading to a comprehensive view of this family of twenty enzymes [1,2]. In addition to their fundamental role in translation, aminoacyl-tRNA synthetases are also involved in other, unrelated noncanonical functions, such as regulation of gene expression, angiogenesis, and cellular signaling [3,4,5]. These secondary functions very often involve their association with cellular partners that are distinct from their regular partners in translation. When mutations affect the functioning of these enzymes, either in their translational or nontranslational functions, they have been associated with diseases in many cases [6,7,8]. Our understanding of the many implications of this family of enzymes thus requires a detailed knowledge of their mode of association with alternative partners.

Here, the analysis of aminoacyl-tRNA synthetase complexes is mainly restricted to the description of the complexes containing several synthetases, and does not address the transient or stable association of a single one of these enzymes with another protein (reviewed in [5]), such as the association of *Escherichia coli* ProRS with YbaK, an editing domain appended in trans to several synthetases [9], of *Methanocaldococcus jannaschii* ProRS with HmdII, an inactive paralog of Hmd dehydrogenase that binds tRNA [10,11], or of *Saccharomyces cerevisiae* SerRS with Pex21p, a peroxisome biogenesis factor that strengthens the interaction of SerRS with its cognate tRNA [12]. During completion of this manuscript, an interesting review describing other complementary aspects of aminoacyl-tRNA synthetase complexes has been published [13].

## 2. Multi-Aminoacyl-tRNA Synthetase Complexes (MARS)

Early works provided some evidence for the existence of complexes containing several aminoacyl-tRNA synthetases in the bacteria *E. coli* [14], in the yeast *S. cerevisiae* [15], or in rat liver [16]. These authors described the occurrence of high-molecular-mass aminoacyl-tRNA synthetases in crude extracts of cells after analysis by chromatography on agarose columns or by centrifugation in sucrose gradients, but also noticed that these assemblies were fragile and highly prone to dissociation. In the light of recent data showing that aminoacyl-tRNA synthetases, and more generally components of the translation apparatus, are able to transiently interact with cellular components such as polysomes or actin filaments [17,18,19], the finding that some components are coeluting as large entities is not sufficient to ascertain that they form complexes. The occurrence of aminoacyl-tRNA synthetases within complexes of defined composition, which are not the result of subcellular interactions with filamentous structures such as mRNA or actin and tubulin polymers and involve direct protein-protein interactions between partners, requires isolation and characterization of the complexes.

Here, the composition of the MARS isolated and characterized in different species is presented, with the aim to identify the rules governing the assembly of these enzymes and the possible involvement of additional factors in this process.

### 2.1. Complexes in Archaea

It was long believed that association between aminoacyl-tRNA synthetases to form complexes is restricted to eukaryotic cells. However, analysis of the structural organization of synthetases in archaea provided compelling evidence for the occurrence of multi-aminoacyl-tRNA synthetase complexes.

**Figure 1:**
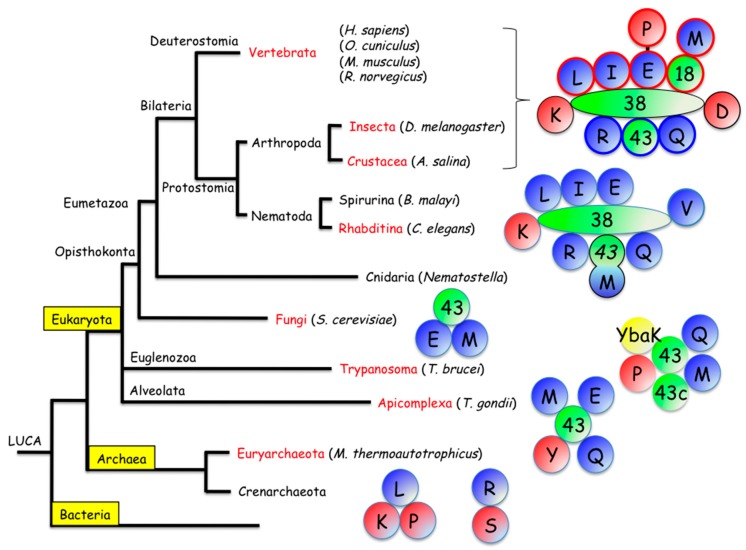
Occurrence of multi-aminoacyl-tRNA synthetase complexes of different compositions throughout the tree of life. A schematic view of the complexes described in Euryarchaeota, Apicomplexa, Trypanosoma, Fungi, Rhabditina, Crustacea, Insecta, or Vertebrata, is shown. The auxiliary proteins known to have a structural role within the complexes are presented in green. Class I synthetases are in blue; class II in red. Synthetases are indicated according to the one letter symbol of their amino acid substrate. The autonomous editing protein YbaK is in yellow. On the right are represented the complexes described in the literature. The complex isolated from vertebrata is composed of a scaffolding protein (p38), that joins sub-complex I (components circled in red), subcomplex II (components circled in blue), LysRS (K) and AspRS (D). In the complex from Rhabditina, the p43 and MetRS (M) proteins are fused in a single polypeptide. The scaffold protein of the complexes from Apicomplexa and Trypanosma have characteristics of p43 from vertebrates.

In the archaea *Methanothermobacter thermoautotrophicum*, a complex containing LeuRS, LysRS and ProRS has been isolated (Figure 1) [20]. The dissociation constants determined for association of LeuRS with LysRS and LeuRS with ProRS were in the micromolar range, suggesting the assembly of a stable complex. Structural mapping of this complex indicated that LeuRS plays the role of a scaffolding protein, the core, *N*-terminal domain of LeuRS binding LysRS and its *C*-terminal domain binding ProRS [21]. In addition, elongation factor EF1A, which forms a ternary complex with GTP and aminoacyl-tRNA to deliver aatRNA to the ribosome, also associates with the CP1 proofreading domain of LeuRS [22]. Stable association (*K*_D_ of 250 nM) of SerRS and ArgRS from the same organism (Figure 1) was also reported to improve their catalytic activity at high temperature [23]. These two synthetases interact with the ribosome in the region of the L7/L12 stalk [24].

In another species of archaea, *Thermococcus kodakarensis*, affinity purification of LeuRS resulted in the isolation of several components of the translation machinery that were identified by mass spectrometry, suggesting that LeuRS forms a complex with TyrRS and ProRS [25]. This complex may also interact with EF1A and ribosomes. However, direct interaction between all these components through protein-protein interaction remains to be established.

A characteristic feature of all the MARS described so far in archaea, is that they do not possess auxiliary proteins involved in their assembly. The scaffold protein of the MARS in *M. thermoautotrophicum* is LeuRS, one the synthetase components [21]. This is a distinctive feature of these complexes, as compared to those found in eukaryotes.

### 2.2. Complexes in Unicellular Eukaryotes

#### 2.2.1. The MARS in *Saccharomyces cerevisiae*

In the yeast *Saccharomyces cerevisiae*, MetRS forms a complex with GluRS via association with Arc1p, a protein homologous to the p43 component of the MARS found in mammals (Figure 1) [26]. Inactivation of Arc1p by gene disruption in yeast is not lethal but results in slow growth. In the MetRS-Arc1p-GluRS complex, Arc1p has two functions. First, it plays a role of cofactor for the two synthetases. The *C*-terminal domain of Arc1p is a tRNA-binding protein. Association of Arc1p with MetRS and GluRS stimulates the tRNA-aminoacylation activity of the two synthetases. The levels of stimulation of aminoacylation activity range from 400-fold [27] to 4-fold [28] for MetRS, and is about 2-fold for GluRS [29] in the presence of Arc1p. In yeast, *S. cerevisiae* Arc1p can be replaced by human p43, a protein that does not interact with *S. cerevisiae* MetRS and GluRS, suggesting that Arc1p is also involved in the sequestration of tRNA in the cytoplasm to increase its local concentration [30]. Second, the intracellular location of the two synthetases rests on the assembly/disassembly of the complex with Arc1p. In the absence of Arc1p, MetRS is redistributed to the nucleus [31,32] and GluRS to the mitochondria where it synthesizes Gln-tRNA^Gln^ via the GatFAB-dependent transamidation pathway [33]. The role of nuclear MetRS remained unclear until the recent discovery that it regulates the expression of genes involved in oxidative phosphorylation in mitochondria, in an unexpected concerted manner between nuclear and mitochondrial genes [32].

*Saccharomyces cerevisiae* is able to grow on either fermentable (glucose) or nonfermentable (ethanol) carbon sources. When cells are switched from fermentation to respiratory metabolism, the oxidative phosphorylation pathway in mitochondria is stimulated to increase ATP production. In particular, the F_1_F_O_ATP synthase complex is overexpressed. This complex is made of several subunits that are either expressed in mitochondria, or are encoded by the nuclear genome. It has been shown that dissociation of the MetRS-Arc1p-GluRS complex is responsible for the synchronized expression of the various subunits of the F_1_F_O_ATP synthase complex [32]. During the transition from fermentation to respiration, expression of Arc1p is downregulated via the Snf1/4 pathway, leading to the release of both MetRS and GluRS from the MetRS-Arc1p-GluRS complex [32]. GluRS is at least in part relocated to the mitochondria in response to a higher demand in the translation of the mitochondria-encoded subunits of the F_1_F_O_ATP synthase complex [33], and MetRS is translocated to the nucleus where it participates in transcription of nuclear ATP synthase genes via a still uncharacterized mechanism [32]. Because MetRS and GluRS are essential enzymes for cytosolic translation, it would be interesting to get more insight on the extent of GluRS and MetRS relocation during the diauxic shift, and on the reverse mechanism after transition from respiration to fermentation growth conditions.

Genetic studies suggested that the *N*-terminal domain is necessary and sufficient for binding the *N*-terminal appended domains of MetRS and GluRS [31]. The crystal structure of two binary complexes made of the N-domain of Arc1p in complex with either the N-domain of MetRS or the N-domain of GluRS, suggested a possible mode of assembly of this complex [34]. The *N*-terminal domains of the three proteins adopt a GST (glutathion S-transferase)-like fold. Whereas classical GST proteins are dimers, Arc1p is a monomer in solution [28]. While the geometry of interaction between Arc1p and MetRS is reminiscent of a canonical GST dimer, Arc1p and GluRS also interact via their GST-like moieties but use different protein interfaces, allowing Arc1p to simultaneously bind MetRS and GluRS [34]. The assembly of the complex solely rests on these interactions, since site-directed mutagenesis of residues located at the interfaces, or deletion of these domains result in cellular relocation of the two synthetases [31,32,35]. Arc1p is a target of Blp1 in yeast, a biotin:protein ligase, but biotinylation of Arc1p at Lys^86^, within its *N*-terminal domain, has no effect on the assembly of the MetRS-Arc1p-GluRS complex [36]. The structure of the ternary complex is highly dynamic in solution, as revealed by small-angle X-ray scattering analyses conducted in the absence or in the presence of tRNA^Met^ and tRNA^Glu^ [37]. The radius of gyration of the ternary MetRS-Arc1p-GluRS complex is of 97 Å, as compared to 60 Å for the pentameric complex obtained after addition of tRNA. This suggests that the activity of Arc1p as a cofactor for binding tRNA to the active site of the two synthetases requires large conformational changes resulting in a compaction of the complex. These data also point to a large flexibility of the complex, a characteristic that hinders its structural study at high resolution.

#### 2.2.2. The MARS in *Toxoplasma gondii*

*Toxoplasma gondii* is an intracellular parasite, a member of the phylum of Apicomplexa, corresponding to unicellular eukaryotes. In Toxoplasma, a protein named Tg-p43 shares about 35% sequence similarity with human p43, especially in its *C*-terminal domain, which is identified as the tRNA-binding EMAPII domain. This observation suggested that a MARS is present in Toxoplasma. Using this p43-like protein as a bait, a complex containing MetRS and GluRS (as found in *S. cerevisiae*), but also GlnRS and TyrRS was isolated (Figure 1) [38]. Similarly to the MetRS-Arc1p-GluRS complex in yeast, the N-domain of Tg-p43 was sufficient to form the complex. Deletion of Tg-p43 is not lethal for Toxoplasma, and does not alter its pathogenicity. Initial electron microscopy imaging of the complex suggests a large degree of flexibility of the particle around a central ring-like core.

#### 2.2.3. The MARS in *Trypanosoma brucei*

*Trypanosoma brucei* is another parasite belonging to the large phylum of flagellate protozoa. TAP-tagged MetRS, ProRS, IleRS and MCP2, a protein sharing similarities with human p43, were copurified with several components of the translation machinery that were identified by mass spectrometry [39]. Globally, three aminoacyl-tRNA synthetases (MetRS, ProRS, GlnRS) could form a core complex with MCP1 and MCP2 (MARS complex-associated proteins 1 and 2), two proteins related to human p43, and MCP3, a protein showing similarities with YbaK, a free-standing editing domain (Figure 1). MCP-2 only contains the *C*-terminal binding domain found in yeast Arc1p and in human p43, and MCP1 also contains the GST-like *N*-terminal domain of full-length Arc1p and p43. AlaRS, TrpRS and AspRS, but also CysRS, GluRS, GlyRS and IleRS could be more loosely associated to the core complex. MCP2 enhances tRNA aminoacylation catalyzed by the associated enzymes, and repression of MCP2 in *T. brucei* reduces parasite growth and infectivity in mice. More detailed biochemical and structural characterization of this MARS would help to clarify its composition, structural organization and function.

### 2.3. The MARS in Eumetazoa

#### 2.3.1. The MARS in *Deuterostomia*

##### Composition of the MARS

The aminoacyl-tRNA synthetase complex that was first isolated is the MARS from vertebrates. Co-isolation of several aminoacyl-tRNA synthetases is invariably observed when synthetases are purified from rat liver, rabbit liver or reticulocytes, sheep liver and human placenta [40,41,42,43,44,45,46,47], or from mammalian cells in culture [48,49]. It contains the nine synthetases specific for the amino acids Arg, Asp, Gln, Glu, Ile, Leu, Lys, Met and Pro (Figure 1). In addition, three additional proteins always copurify with the particulate synthetases, p18 [50], p38 [51] and p43 [52]. One of the synthetase components contains two aminoacyl-tRNA synthetases fused on a large, single polypeptide chain of 171 kDa, corresponding to glutamyl-prolyl-tRNA synthetase (GluProRS) [53,54]. The two-synthetase domains are separated by a linker region made of three repeated sequence motifs of about 50 amino acids, named the WHEP domains because they were discovered in human TrpRS (W), HisRS (H), and GluProRS (EP). Their occurrence in human MetRS and GlyRS was also reported later on. This polypeptide arose from a gene fusion event [55,56] occurring more than 1 billion years ago in Eumetazoa, after divergence from Fungi [57]. This complex contains monomeric (ArgRS, GlnRS, IleRS, LeuRS, MetRS) or dimeric enzymes (AspRS, GluProRS, LysRS) for a mass of about 1.5 MDa determined by SEC-MALS analysis [58]. Among these components, the same gene encodes two distinct forms of ArgRS, GluRS and LysRS. In addition to the 74 kDa species of ArgRS present in the MARS, another form of 60 kDa is produced by alternative translation initiation on a second ATG on the same mRNA [59,60]. ArgRS from the MARS delivers Arg-tRNA for translation, and Arg-tRNA produced from the free form is believed to deliver tRNA for arginylation of proteins in the ubiquitin-dependent degradation pathway [61,62]. Two copies of GluProRS are present in the MARS [58], and a truncated form of GluProRS containing only the GluRS moiety was identified [63]. This GluRS-only species is expressed from the same gene after polyadenylation-directed conversion of a Tyr codon to a stop codon. It is not known whether this GluRS participates in protein translation in the cytoplasm. Concerning LysRS, a single gene encodes the cytoplasmic and mitochondrial enzymes which are produced by alternative splicing of exon 2 of the KARS gene [64]. The mRNA with a deletion of sequences encoded by exon 2 expresses the cytoplasmic species of LysRS produced by translation initiation in mRNA sequences encoded by exon 1. The cytoplasmic species of LysRS is the only one to be present in the MARS complex [65]. The reason for the selection of these nine synthetases and only those nine is unclear but a correlation with the size of their amino acid substrates [66] or with their connection with intermediates of the citric acid cycle [67] has been proposed.

The three auxiliary proteins have an essential structural role for the assembly of the MARS, and also fulfill important functions in the translation machinery. The p38 protein of the MARS is a dimeric protein made of 320 amino acid residues with no homolog in yeast, bacteria and archaea. It has the potential to interact with several components of the MARS and was thus identified as the scaffold protein of the complex [51]. Its *C*-terminal moiety is a GST-like domain, and its *N*-terminal region contains a leucine zipper motif [51,68]. A splice variant of p38 with a deletion of exon 2, expressing a protein with a deletion of the leucine zipper region, does not associate within the MARS and promotes tumorigenesis via degradation of p53 [69]. Assembly of the MARS is severely impaired after shRNA-mediated knockdown of p38 in HeLa cells [70], or after introduction of mutation within the structural gene of p38 in mice [71]. In neurons, a balanced expression of p38 with other components of MARS is regulated by ubiquitin-dependent degradation of p38, which involves Parkin, an E3 ubiquitin-protein ligase. Abnormal accumulation of p38 in dopaminergic neurons is linked to Parkinson’s disease, due to progressive neurodegeneration [72,73,74]. The p18 component is a small protein of 174 amino acid residues [50]. The crystal structure reveals a GST-like fold similar to that observed for the yeast Arc1p component of the MetRS-Arc1p-GluRS complex [75]. It promotes association of MetRS within the MARS [70] and is also involved in transfer of Met-tRNA_i_^Met^ to eukaryotic initiation factor 2 (eIF2) [76]. The p43 component has homology with yeast Arc1p [52]. The native protein has a strong tRNA-binding capacity (*K*_D_ of 0.2 µM) that is lost upon cleavage with caspase 7 which generates endothelial-monocyte-activating polypeptide II (EMAPII) [77], an inflammatory cytokine released under apoptotic conditions [78]. The EMAPII domain is similar to bacterial tRNA-binding protein Trbp111 [79] and forms an oligonucleotide-binding (OB) fold [80,81]. Its *N*-terminal moiety anchors the procytokine to the MARS and is required for association of GlnRS and ArgRS to the complex [70]. The tRNA-binding domain of p43 is supposed to act as a cofactor *in trans* for the binding of tRNA by one or several synthetases of the MARS. A role of p43 in the delivery of tRNA to the ArgRS component of MARS was proposed [82], but another study did not confirm these data [83]. Interestingly, two p43 proteins of different lengths are encoded by the same gene and produced by the same mRNA [84]. Translation initiation from two in-frame AUGs generates mitochondrial and cytoplasmic forms of p43. The longest translation product contains nine additional *N*-terminal amino acid residues, which correspond to a mitochondrial targeting sequence (MTS). The function of p43 in mitochondria is not known.

##### Assembly of the MARS

**Figure 2:**
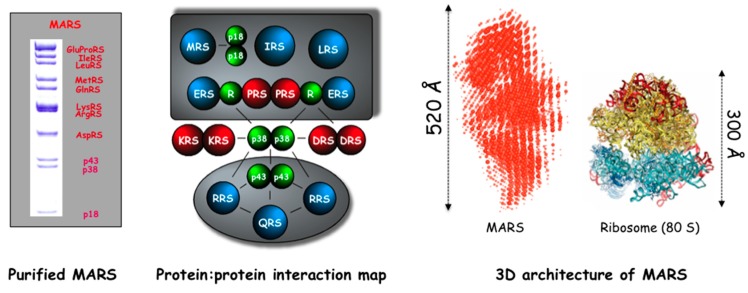
Composition, structural organization and 3D-architecture of the MARS. **Left**: SDS-PAGE analysis of the MARS from rabbit; **Middle**: protein:protein interaction map of the MARS, showing two subcomplexes linked by a scaffold protein, p38; **Right**: low resolution envelope of the MARS determined by SAXS (Small Angle X-ray Scattering). The crystal structure of the 80S ribosome is shown for comparison.

In vertebrates, highly purified preparations of the MARS contain eleven polypeptides with molecular masses ranging from 171 kDa for GluProRS to 20 kDa for p18 (Figure 2) [58]. Structural mapping of the complex to determine topological relationships between the various components has been conducted by different approaches: reverse chemical crosslinking [85], extensive two-hybrid searches for pairs of interacting partners [51,86], pull-down experiments of native proteins, of truncated derivatives or of fragments to identify the regions of the two partners involved in protein–protein interactions [83,87,88,89], assembly of subcomplexes with purified individual components [89], or knockdown of the non-essential p18, p38 and p43 proteins in cultured cells that induced perturbations of the assembly of the complex [70]. Although many of the reported data were contradictory, a general scheme of complex assembly could be proposed, which reconciles most of the published data (Figure 2). The MARS is made of two subcomplexes linked by the p38 scaffold protein [51,70,71]. Sub-complex 1 contains GluProRS, IleRS, LeuRS to which MetRS is also associated via the auxiliary protein p18. Sub-complex 2 associates ArgRS and GlnRS with p43. LysRS and AspRS are direct interactors of p38. The complete network of interactions results in a stable particle that cannot be easily dissociated under nondenaturing conditions. Binding affinities in the range of 0.3 nM to more than 100 nM were determined for association of LysRS with 38, or p43 with ArgRS, respectively [89].

Association of synthetases to the MARS either involves the appended eukaryote-specific domains or the core domain of the components. The GST-like *N*-terminal appended domain of MetRS that interacts with p18 [90,91,92], the leucine-rich *N*-terminal appended domain of ArgRS that interacts with p43 [83], the *C*-terminal appended domains of LeuRS [87] and IleRS [88], and the GST-like *N*-terminal appended domain of GluProRS, are required for complex assembly. Concerning LysRS, AspRS and GlnRS, the core domains of the synthetases are believed to be involved in complex assembly [93,94]. Only few structural data are available at high resolution to describe the protein:protein interaction sites. LysRS associates with the very *N*-terminal region of p38 [51]. The crystal structure of a complex made of a *N*-terminally truncated form of LysRS and of a *N*-terminal peptide of p38 reveals the mode of association of these two proteins [95]. The site of interaction with p38, which identifies the site of interaction within the MARS, is located underneath the LysRS dimer, leaving the other side fully accessible for binding and aminoacylating tRNA. The solution structure of the leucine zipper of p38, which interacts with the leucine zipper of p43, has also been reported but does not provide precise structural information about organization of the p38:p43 complex [68].

The assembly of subcomplex II of MARS has been extensively studied. The *N*-terminal appended domain of ArgRS is essential for its assembly within the MARS [60,61]. It mediates its association with p43 [83]. ArgRS and p43 are required for association of GlnRS to subcomplex II [89]. The crystal structure of subcomplex II reveals that the *N*-terminal domain of ArgRS is a long α-helix, which forms a coiled-coil with the *N*-terminal helix of p43, and interacts with the catalytic core of GlnRS [96]. This first high-resolution structure of a subcomplex of MARS, showing an elongated arrangement (100 Å in length), is a significant breakthrough in the field, but many questions are left open. Only one molecule of each ArgRS, GlnRS and p43 are present in the asymmetric unit of the crystal. The hexameric complex deduced from a twofold symmetry operation could represent the native structure of subcomplex II, but this conformation does not allow access of tRNA to the catalytic core of ArgRS. This suggests that activity of ArgRS within the complex requires a large conformational change. Interaction between the long *N*-terminal helical domain of ArgRS and p43 is also supposed to be essential for the activity of ArgRS [96], but earlier results showed that full-length ArgRS and a truncated derivative lacking this *N*-terminal domain have similar kinetic parameters in the tRNA aminoacylation reaction [97].

Low-resolution models of the MARS, obtained by electron microscopy and three-dimensional reconstructions [98] or by small-angle X-ray scattering (SAXS) in solution (Figure 2) [58], reveal a large particle with external dimensions ranging from 25 nm × 30 nm × 23 nm [98] to an elongated molecule of about 50 nm in length [58]. As discussed in this paper, the model of MARS deduced from electron microcopy analyses may represent only a subset of the particle. The multi-armed shape of MARS observed by SAXS certainly reflects the flexibility of the different domains of the complex. This non-compact structural organization of MARS could be appropriate to allow large structural rearrangements upon tRNA binding, as observed for the yeast MetRS-Arc1p-GluRS complex [37]. This open structure may also favor efficient release of the components that have been reported to fulfill non-canonical functions after dissociation from the complex. It would be now interesting to see how the crystal structure of subcomplex II could be fitted into the low-resolution envelope of MARS described by SAXS.

#### 2.3.2. The MARS in Protostomia

##### Arthropoda

The MARS was described in two members of the Arthropoda family (Figure 1), in the Insecta *Drosophila melanogaster* [99] and in the Crustacea *Artemia salina* [100]. The complex purified from the fly *D. melanogaster* contains the same nine aminoacyl-tRNA synthetases and the same three auxiliary proteins, as found in mammals [99]. Its structural organization was not analyzed, but is believed to be very close to that reported for the MARS in *Deuterostomia*. The main differences concern GluProRS, which contains six WHEP domains instead of three in the inter-synthetase region, and MetRS, which contains three WHEP domains appended at the *C*-terminus instead of one. The p43, p38 and p18 proteins share 43%, 21% and 30% identity with the corresponding proteins from human MARS. Because the same MARS was found in Deuterostomia, represented by the MARS isolated from mammals, and in Protostomia, represented by the MARS from the fly *D. melanogaster*, the two branches of Bilateria, it was tempting to speculate that this type of MARS was already present at the origin of Bilateria.

##### Nematoda

**Figure 3:**
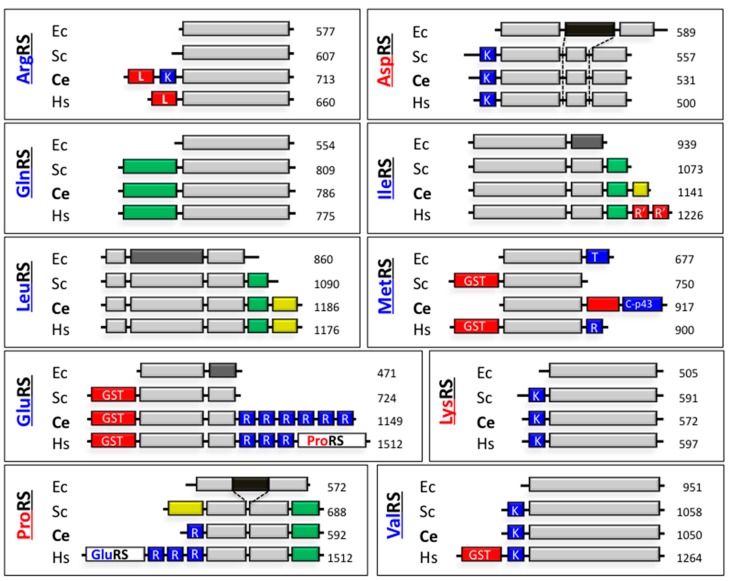
Schematic comparison of ten aminoacyl-tRNA synthetases from *H. sapiens* (Hs) with their homologs in *E. coli* (Ec), *S. cerevisiae* (Sc) and *C. elegans* (Ce). Conserved parts are shown in light grey with poorly conserved regions in medium grey. Dark grey boxes represent *E. coli* specific domains. The number of amino acid residues per polypeptide chain is indicated. Eukaryote specific domains, conserved from yeast to human, are shown in green. Appended domains found only in one or two species are in yellow. Protein–protein and protein–RNA interaction domains are shown in red and blue, respectively: L—leucine-rich domain; GST—glutathion *S*-transferase-like domain; R'—repeated sequence found in human IleRS; K—lysine-rich domain; R—repeat (WHEP) domain. The names of class I aaRS are highlighted in blue, of class II in red.

Nematoda is one of the other main branches of Protostomia (Figure 1). The MARS from *Caenorhabditis elegans*, a member of the Rhabditina sub-branch of Nematoda has been isolated. Aminoacyl-tRNA synthetases from the worm *C. elegans* display a high level of similarity with the human counterparts. Sequence comparison of the synthetases from the MARS of human with homologous proteins from *C. elegans* suggested that some of these enzymes had a high probability to be found in a MARS, whereas some other did not show characteristics of the proteins associated in the human MARS (Figure 3). The core region of the synthetases, corresponding to the catalytic and anticodon-binding domains, which share from 47% identities for ArgRSs to 59% identities for AspRSs, are generally well conserved between *C. elegans* and human, but eukaryote-specific appended domains are remarkably distinct for some pairs of synthetases [101]. AspRS, GlnRS, LeuRS and LysRS from *C. elegans*, which display an extensive global conservation with their human homologs were likely to be found in the MARS of *C. elegans*, together with ArgRS which displays the leucine-rich *N*-terminal extension involved in the assembly of human ArgRS in the MARS. On the contrary, the possible association of IleRS, MetRS, GluRS and ProRS in a complex in *C. elegans* was questionable. IleRS from *C. elegans* does not share the two *C*-terminal repeated domains of human IleRS; MetRS in *C. elegans* has a very distinct structural organization, without a *N*-terminal GST-like domain and with a p43-like *C*-terminal domain [102]; and GluRS and ProRS do not form a fused protein in *C. elegans* (Figure 3). In addition, no protein homologous to p18 or to p43 could be identified in *C. elegans*. Only a very putative p38-like protein with 16% identities with human p38 was reported.

Surprisingly, the MARS isolated from *C. elegans* contains seven of the nine synthetases found in the human MARS, namely ArgRS, GlnRS, GluRS, IleRS, LeuRS, LysRS and MetRS, but does not contain AspRS and ProRS [101]. The p38-like protein is essential for the assembly of the synthetases. In a mutant strain of *C. elegans* with a deletion of the 67 *C*-terminal residues of p38, several synthetases are released from the complex [101]. Association of MetRS to this complex is not mediated by association *in trans* with an auxiliary protein, as in the case of p18 in the human MARS, but by fusion *in cis* of a p43-like domain, suggesting that *bona fide* p43 and MetRS genes fused in *Rhabditina*. At the same time, a fission event occurred in the GluProRS gene [55], leaving only its GluRS moiety attached to the complex via its *N*-terminal GST-like domain.

**Figure 4:**
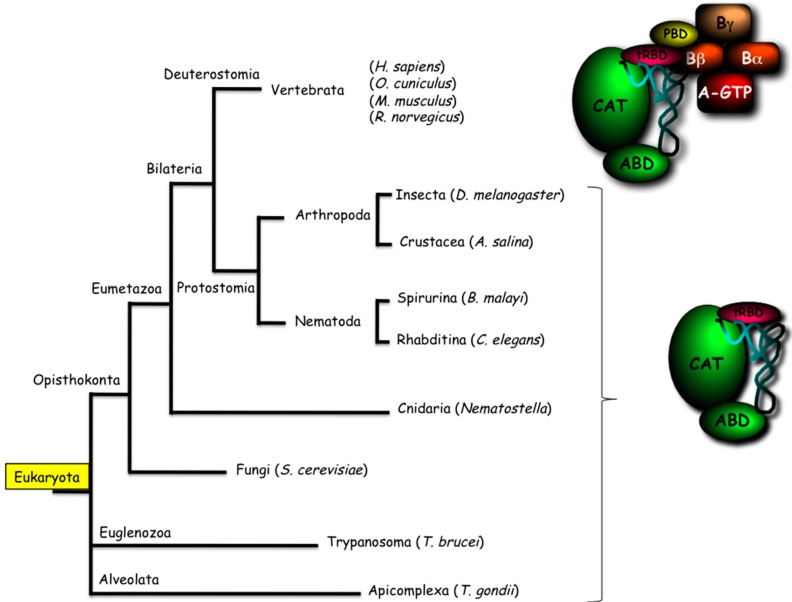
Occurrence of ValRS:EF1A:EF1B complexes or of “free” ValRS in Eukaryota. The conserved catalytic (CAT) and anticodon-binding (ABD) domains are in green. The appended tRNA-binding domain (tRBD) is indicated in red, the protein-binding domain (PBD) only recovered in Deuterostomia, required for association with elongation factor 1, is in yellow.

Unexpectedly, ValRS is associated in the MARS complex of *C. elegans*. This enzyme shares the *N*-terminal tRNA-binding domain found in yeast and human ValRS, but does not possess the GST-like *N*-terminal domain of the human enzyme (Figure 3). In Vertebrata, ValRS forms a complex with elongation factor EF1A, and with the three subunits of its guanine nucleotide exchange factor, EF1Bα, EF1Bβ and EF1Bγ [103,104,105]. The *N*-terminal GST-like domain of ValRS is indispensable for its interaction with the EF1Bβ subunit of the EF1 complex [106]. Analysis of ValRS sequences in Eukaryota reveals that the presence of this GST-like protein-binding domain (PBD), and thus the propensity of ValRS to associate with the EF1A/EF1B complex, could be restricted to the branch of Deuterostomia from Bilateria (Figure 4). In the absence of this domain, association of ValRS with the MARS does not appear to be a general feature of ValRS since no stable association of ValRS was reported with other MARS, especially in the sister group of Arthropoda. Therefore, sequence-based analysis of the synthetases is certainly not sufficient to predict with high confidence their ability to form supramolecular complexes with other synthetases, as in the case of the MARS, or with other components of the translation apparatus such as EF1A/EF1B.

## 3. Integration of the MARS in Cellular Homeostasis

### 3.1. Integration of the MARS in Translation Apparatus

Association of the MARS with elongation factor EF1A and with ribosomes/polysomes has often been reported. The multisynthetase complexes described in archaea have been suggested to interact with EF1A and with ribosomes to facilitate cycling of tRNAs in translation [22,24,25]. In eukaryotes, interaction of the MARS with EF1A, ribosomes and polysomes has also been reported [17,107,108]. Association of components of the translation machinery with the actin filamentous network has been reported [17,109]. The working model assumes that tRNA is never free in the cytoplasm of eukaryotic cells, but is vectorially transferred from the synthetase, to elongation factor EF1A, to ribosome, and back to the synthetase [19,110] suggesting that the process of tRNA cycling in translation is processive [111]. This implies that stable and transient protein:protein, and protein:nucleic acid interactions are essential to ensure proteostasis.

### 3.2. Role of the MARS in Regulation of Other Cellular Functions

In addition to their essential role in translation, several components of the MARS are also involved in nontranslational functions, which were recently described in several excellent reviews [6,7,8]. Only a few examples are summarized below. These noncanonical functions are related to cell signaling and control of cellular homeostasis, and especially to the regulation of fundamental physiological processes such as inflammation, angiogenesis and tumorigenesis. Association/dissociation of components of the MARS could be a means to regulate in space and in time their activity in translational or nontranslational processes [112].

In some cases it has been clearly shown that dissociation from the complex is related to a gain of new functions. After phosphorylation on Ser^207^ in stimulated mast cells, LysRS dissociates from the MARS, translocates to the nucleus and stimulates transcription of genes involved in the immune response via activation of microphtalmia-associated transcription factor MITF [95,113,114]. This transcriptional function of a synthetase rests on the ability of LysRS to synthesize diadenosine tetraphosphate (Ap_4_A) when, in the absence of tRNA, aminoacyl-adenylate can react with a second molecule of ATP [115]. Several aminoacyl-tRNA synthetases from bacteria to mammals, in addition to LysRS, have the ability to synthesize Ap_4_A, which suggests that they may be involved in the regulation of various cellular processes. After phosphorylation of the WHEP domains in IFNγ-stimulated cells, GluProRS dissociates from the MARS, and associates with components of the GAIT (γ-interferon activated inhibitor of translation) complex to induce translational silencing of inflammation-related mRNAs [116,117]. Laminin is a major constituent of the extracellular matrix that interacts with receptors and induces cell migration. After laminin stimulation, LysRS is phosphorylated at Thr^52^ by p38 mitogen-activated protein kinase (MAPK), translocates to the plasma membrane, interacts with the 67LR receptor, and promotes metastasis [118]. Cleavage of the p43 component of the MARS by caspase 7 [77] releases its *C*-terminal domain known as EMAP II (endothelial monocyte-activating polypeptide II) a tumor-derived cytokine that regulates angiogenesis [78,119].

Some components of the MARS are thought to regulate other cellular functions, but it is not clear whether the MARS-associated or -dissociated form of the protein is involved, or the mechanisms of complex-release are not determined. For instance, LeuRS has been reported to be an important regulator of one of the major pathway of cellular homeostasis through the leucine-dependent control of mTORC1 activity [120], but the relationship between MARS-LeuRS and mTORC1-related LeuRS is not known. LeuRS was described to act as a GTPase-activating protein for Rag GTPases that activate mTORC1 in response to amino acid availability [120], but these effects were not recapitulated in another study [121]. GlnRS may have an anti-apoptotic function through its glutamine-dependent interaction with apoptosis signal-regulating kinase 1 (ASK1), but the molecular species of GlnRS involved in this pathway is not known [122].

## 4. Concluding Remarks

### 4.1. Origin of the MARS of the Eukaryotic-Type

In archaea, the multi-synthetase complexes are characterized by the absence of non-synthetase scaffold proteins. The majority but not all of the components found so far associated within the complexes described in Fungi, Trypanosoma or Apicomplexa are also recovered in Eumetazoa, and contain accessory proteins with scaffolding properties. The finding that composition of the MARS recovered in different species is not the result of serial addition of components suggests that at least during the early steps of emergence, evolution of MARS followed a non-linear scheme of trials and errors before establishment of more universal rules of assembly, as observed in Bilateria. However, even in Bilateria the complex isolated in *C. elegans* casts some doubts about the universality of the MARS in higher eukaryotes. However, the finding that essentially the same MARS is present in Deuterostomia and Protostomia (Arthropoda) suggests that it pre-existed in the last common ancestor of Bilateria. The atypical MARS isolated from *C. elegans* could be the result of genome rearrangements occurring in a limited set of Nematoda belonging to the Rhabditina branch, as also proposed for evolution of GluProRS [55,57]. In *Haemonchus contortus*, *Necator americanus* or *Ancylostoma ceylanicum*, three nematodes from the Rhabditina branch of Nematoda, GluRS and ProRS are encoded by distinct genes, as in *C. elegans*. It could be the result of a fission event of the GluProRS gene appearing in a limited set of species. Indeed, in the two very closely related nematodes from the Spirurina branch of Nematoda, *Brugia malayi* and *Loa loa*, GluRS and ProRS are fused on a single gene, as also observed in *Trichuris suis* or *Trichinella spiralis*, two nematodes from the more distantly related branch of Enopla. The fission of GluProRS and MARS rearrangement could be linked. Because a *bona fide* GluProRS was also observed in the cnidarian sea anemone *Nematostella vectensis* [55], this suggests that a MARS similar to that observed in Deuterostoma could exist in Cnidaria, which would locate the origin of the eukaryotic-type MARS near the origin of Metazoan.

### 4.2. Understanding the Balance between Translational and Non-Translational Functions

The balance between their translational and noncanonical functions very often involves dissociation of components of the MARS and their association with alternative partners. The knowledge of the protein interfaces involved in the different facets of their activity is of fundamental importance as far as their connection with various physiological disorders and diseases is concerned. Indeed, it should be kept in mind that an intricate interaction network makes it more difficult to design molecules capable of inhibiting a single pathway.

Cellular homeostasis requires a tight control of the various functions of aminoacyl-tRNA synthetases in translational and nontranslational processes. Obviously, overexpression of a synthetase or drastic perturbation of cellular equilibrium after siRNA silencing of these essential proteins, are likely to induce responses which may not be physiologically relevant. 

LysRS is the component of the MARS for which most noncanonical functions have been described in human [7]. When it accomplishes its translational function, the catalytic domain of LysRS interacts with p38 within the MARS. On the other hand, LysRS dissociates from the cytoplasmic MARS to regulate the activity of the transcription factor MITF in the nucleus [114]. LysRS has been reported to interact with several proteins: with synthenin-1 which modulates the activity of LysRS [123], with the laminin receptor which controls the stability of the receptor [118], and with the Gag polyprotein of HIV-1 for packaging tRNA_3_^Lys^ into the virions [124]. The mitochondrial and cytoplasmic species of LysRS are encoded by the same gene and share the same catalytic and anticodon-binding domains. The conserved region of mitochondrial LysRS interacts with mutant SOD1 in some cases of amyotrophic lateral sclerosis [125], or with the GagPol polyprotein of HIV-1 to package tRNA_3_^Lys^ into viral particles [94]. It is not known whether the same surface area of LysRS is involved in the interaction with p38 and with all these secondary partners. LysRS can bind non competitively to MARS and to GagPol, suggesting that these binding sites are independent [94]. In that case, it is conceivable to isolate an inhibitor of the association between LysRS and GagPol that would not impair association of LysRS into the MARS and would not be toxic for the translation process. The LysRS:p38 interface has been described at the atomic level, from the co-crystal structure of LysRS with a non-structured peptide corresponding to the *N*-terminus of p38. It remains to be established whether association of LysRS with the native, full-length scaffold protein will reveal a similar interaction pattern. 

### 4.3. Perspectives

During the past few years, understanding the function of aminoacyl-tRNA synthetases appeared to be a task much more complicated than previously anticipated due to the numerous secondary, noncanonical functions that are performed by this family of enzymes. Association and dissociation of the components of the MARS seems to be an important checkpoint for many cellular pathways. The recent finding that splice-variant synthetases may fulfill functions independently of their primary role in translation, also unexpectedly expands the sphere of influence of this family of enzymes [126].
